# A Different Type of Tension Headache: A Case Report of Traumatic Tension Pneumocephalus

**DOI:** 10.21980/J8DH0G

**Published:** 2021-04-19

**Authors:** Travis P Sharkey-Toppen, Dominique I Dabija, Christopher San Miguel

**Affiliations:** * The Ohio State University Wexner Medical Center, Department of Emergency Medicine, Columbus, OH

## Abstract

Tension pneumocephalus is a rare neurosurgical emergency requiring cranial decompression to prevent permanent neurological injury, herniation, and death. In this report, we present a trauma patient struck by a knife in the forehead who presented with agitation and vomiting. Imaging ultimately revealed a minimally displaced frontal sinus fracture through both the anterior and posterior walls of the sinus. This disruption, acting as a ball-valve for air entry into the cranium, resulted in extensive pneumocephalus and eventual tension physiology. The patient required immediate neurosurgical decompression. This case illustrates both the importance in recognition of this uncommon pathology as well as the need for rapid reassessment with clinical changes in trauma patients.

**Topics:**

Tension pneumocephalus, facial fracture, head trauma.

**Figure f1-jetem-6-2-v13:**
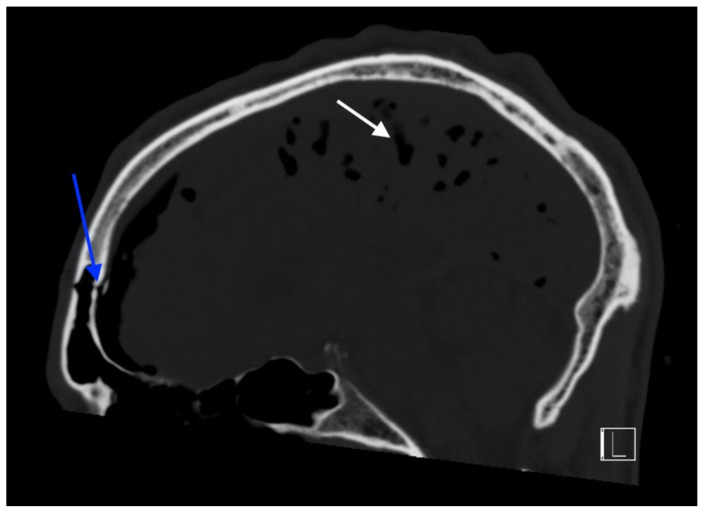


**Figure f2-jetem-6-2-v13:**
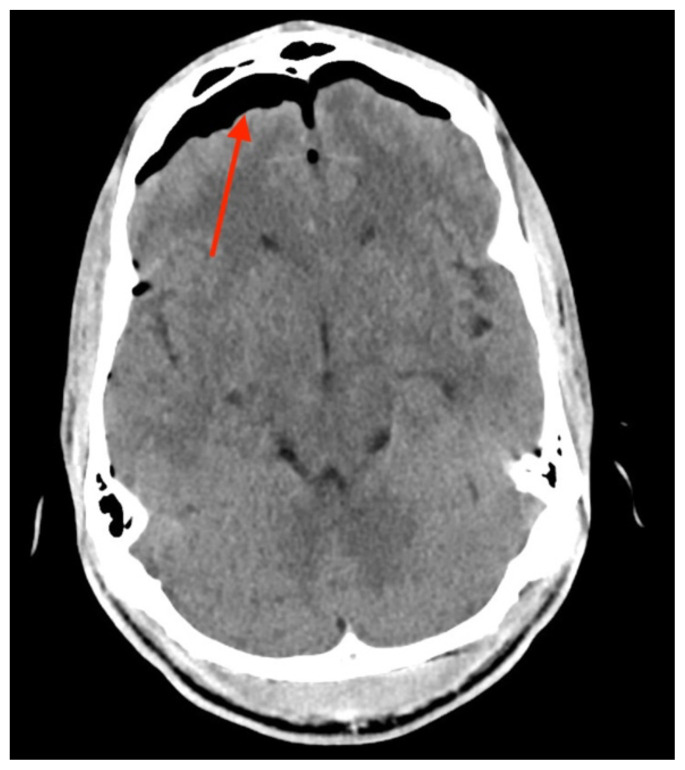


## Brief introduction

[Fig f1-jetem-6-2-v13][Fig f2-jetem-6-2-v13]Pneumocephalus, the introduction of air within the cranium, can occur as the result of traumatic injury or as a postsurgical complication.^1^ Although pneumocephalus is common after neurosurgical intervention, it rarely progresses to tension physiology.^2^ The diagnosis of tension pneumocephalus (TP) requires clinical findings of neurological deterioration secondary to increased intracranial pressure (ICP) from increased volumes of entrapped air; this is more commonly seen after traumatic introduction of air into the cranium.^3^,^4^ Tension pneumocephalus presents similarly to other forms of mass effect with headache, nausea, vomiting, depressed level of consciousness, and focal neurological deficits. It can also trigger the Cushing reflex, causing bradycardia, irregular respirations, and widened pulse pressure.^5^

## Presenting concerns and clinical findings

A 33-year-old male without significant past medical history presented to the emergency department (ED) after a reported assault. The patient drove himself to the ED after reportedly being struck by a knife to his forehead. The patient stated that he was suddenly attacked by a stranger with a knife and hit once against his forehead, resulting in a laceration. He denied any loss of consciousness or additional areas of injury. On primary survey, the patient was hemodynamically stable, alert, oriented, and following commands with a single laceration to his mid-forehead approximately 1-cm in length and without active bleeding. During the secondary survey, the patient became profusely diaphoretic and had a single episode of emesis. At that time, the decision was made to obtain computed tomography (CT) of his head with concern for intracranial injury. Other than agitation, his neurological exam was normal.

## Significant findings

CT head without contrast demonstrated a minimally displaced fracture of the frontal sinuses at the midline underlying his known laceration that involved the anterior and posterior tables of the calvarium. This is seen on the sagittal view and indicated by the blue arrow. There was a small volume of underlying subarachnoid hemorrhage along the falx. There was also extensive pneumocephalus most pronounced along the bilateral anterior frontal convexity associated with the frontal sinus fracture, seen on the axial image and indicated by the red arrow. This pattern of air is commonly referred to as the “Mount Fuji” sign.^6^ Other intracranial air can also be seen on the sagittal image and is indicated by the white arrow.

## Patient course

During his ED course, the patient was transferred to a level 1 trauma center for neurosurgical evaluation and expected decompression. The patient received a tetanus booster, was started empirically on ampicillin-sulbactam for his open facial fracture, and his laceration was closed to help minimize air entry while awaiting definitive management. No additional injuries were identified on complete traumatic evaluation, which included plain film x-rays of his chest and pelvis as well as CT of his cervical spine. During evaluation, the patient had notable bradycardia and worsening headache while seated upright, prompting concern for worsening mass effect. The patient underwent neurosurgical decompression and dura repair, hematoma evacuation, pericranial flap and frontal sinus obliteration to relieve the tension physiology in addition to open reduction and internal fixation of his facial fractures. He was started on a prophylactic anti-epileptic, levetiracetam. The patient was discharged on hospital day 3 with directions to continue prophylactic levetiracetam for seizure prevention as well as a 28-day prophylactic antibiotic regimen with amoxicillin-clavulanate. He demonstrated no neurological deficits at time of discharge and remained without seizure, wound infection, or neurological deficit at his follow-up visit two weeks later. He was instructed to continue home wound care and antibiotics with continued follow-up after completion of the antibiotic regimen.

## Discussion

Benign pneumocephalus, seen most commonly as a post-operative complication, may typically be treated conservatively and rarely progresses to TP.^7^,^8^ Conservative treatment includes oxygen therapy, maintaining supine or Trendelenberg position, prophylactic antibiotics for associated fractures, and frequent monitoring for the evidence of acute worsening or tension physiology.^3^

Tension pneumocephalus is a rare presentation for blunt and penetrating traumatic injuries. Although commonly found to have the “Mount Fuji” sign on CT imaging of the head, TP is a clinical diagnosis with signs and symptoms of neurologic deterioration or increased ICP in addition to known pneumocephalus on imaging.^3^,^6^ Traumatic TP results from a discrete calvarium defect or fracture that acts as a ball-valve to allow for intracalvarium air entry that becomes trapped, increasing intracranial pressure. If left untreated, this can progress to ischemic injury, herniation, and even death, thus requiring emergent neurosurgical intervention.

It is imperative to recognize early signs of increased pressure including headache, nausea, and vomiting. More significant changes such as altered level of consciousness, focal neurological deficits, or evidence of Cushing reflex should further raise suspicion for mass effect or increased ICP, which is common not only in TP but also the more likely diagnosis of intracranial hemorrhage.^5^

Therapies focus on relieving pressure caused by trapped air, including needle aspiration, burr holes, craniotomy, ventriculostomy, and closure of dural defects.^3^ Unlike typical ICP treatments, temporizing measures should avoid hyperventilation that may lead to decreased cerebral blood flow, and thus increased subdural space available for air entrapment.^8^ If intubation is necessary, high airway pressures should also be avoided because they may increase intrathoracic pressures and impede cerebral venous return, worsening intracranial pressures.^8^ Normobaric hyperoxia by intubation or high-flow nasal cannula has also been shown to facilitate faster resorption of air.^9^,^10^

## Supplementary Information








