# Supplementary figures and images for: A Different Type of Tension Headache: A Case Report of Traumatic Tension Pneumocephalus
Source: J Educ Teach Emerg Med. 2021 Apr 19;6(2):V13–5. doi: 10.21980/J8DH0G (PMC10332778; doi:10.21980/J8DH0G)

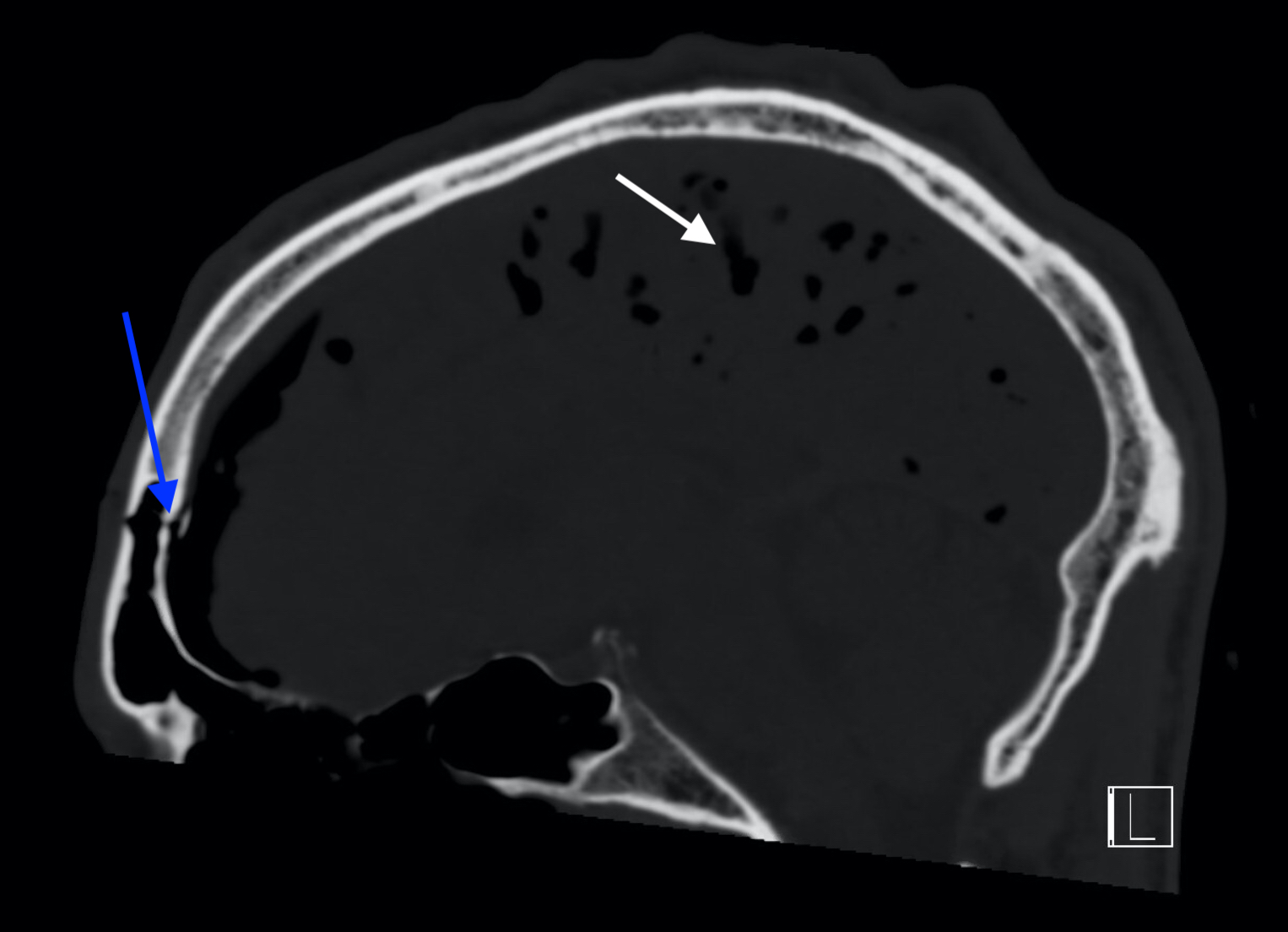

Supplement: Supplementary file 1 [file jetem-6-2-v13-supp1.jpg]

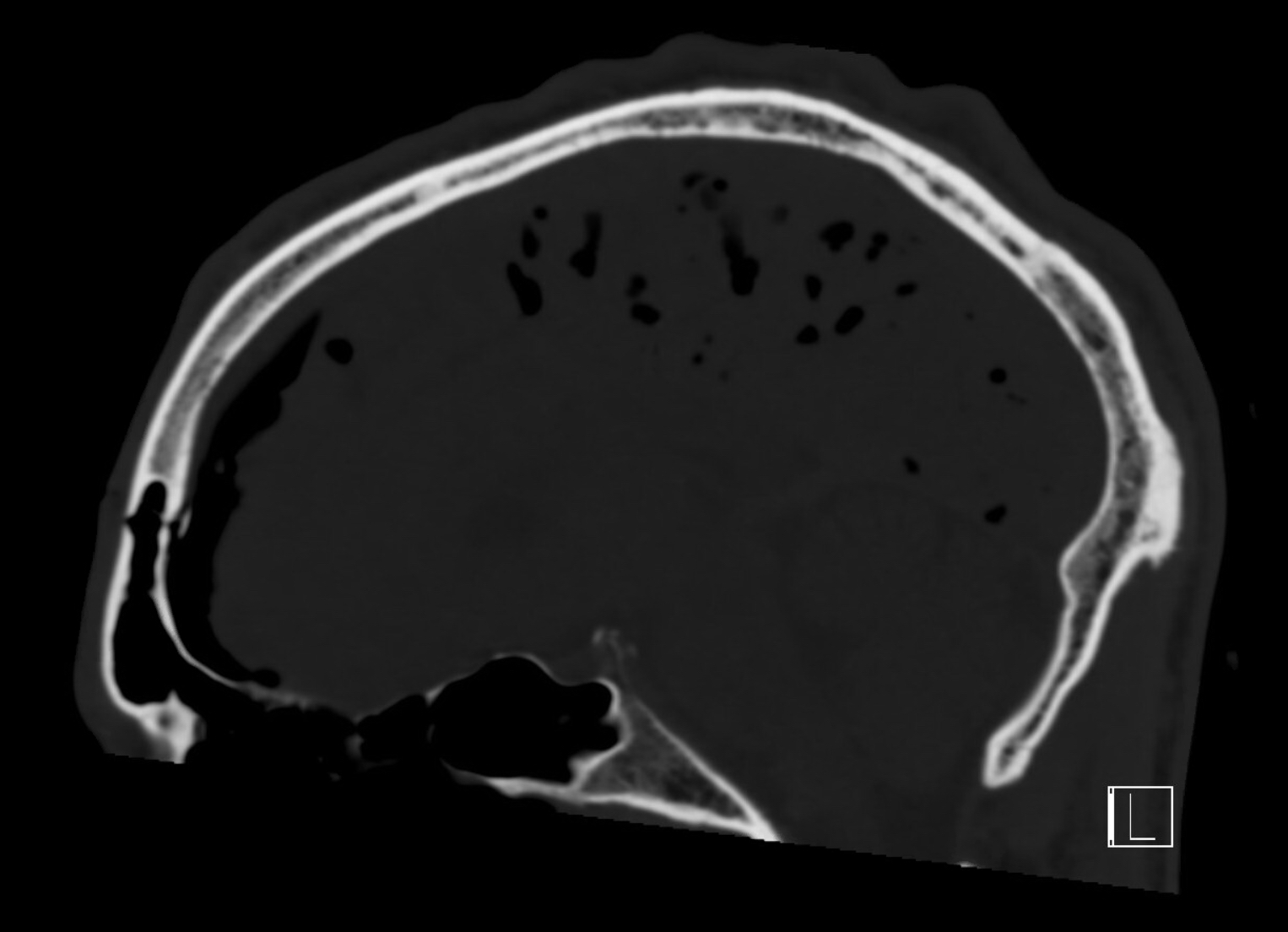

Supplement: Supplementary file 2 [file jetem-6-2-v13-supp2.jpg]

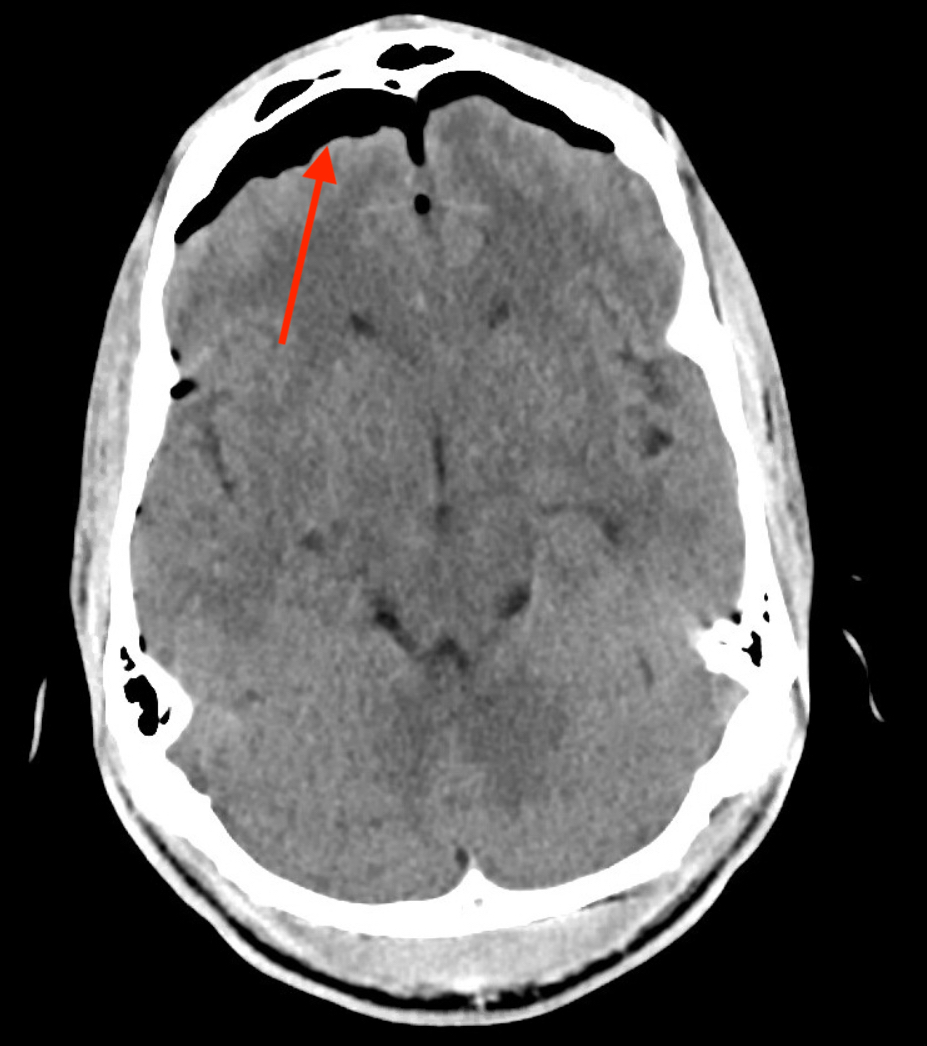

Supplement: Supplementary file 3 [file jetem-6-2-v13-supp3.jpg]

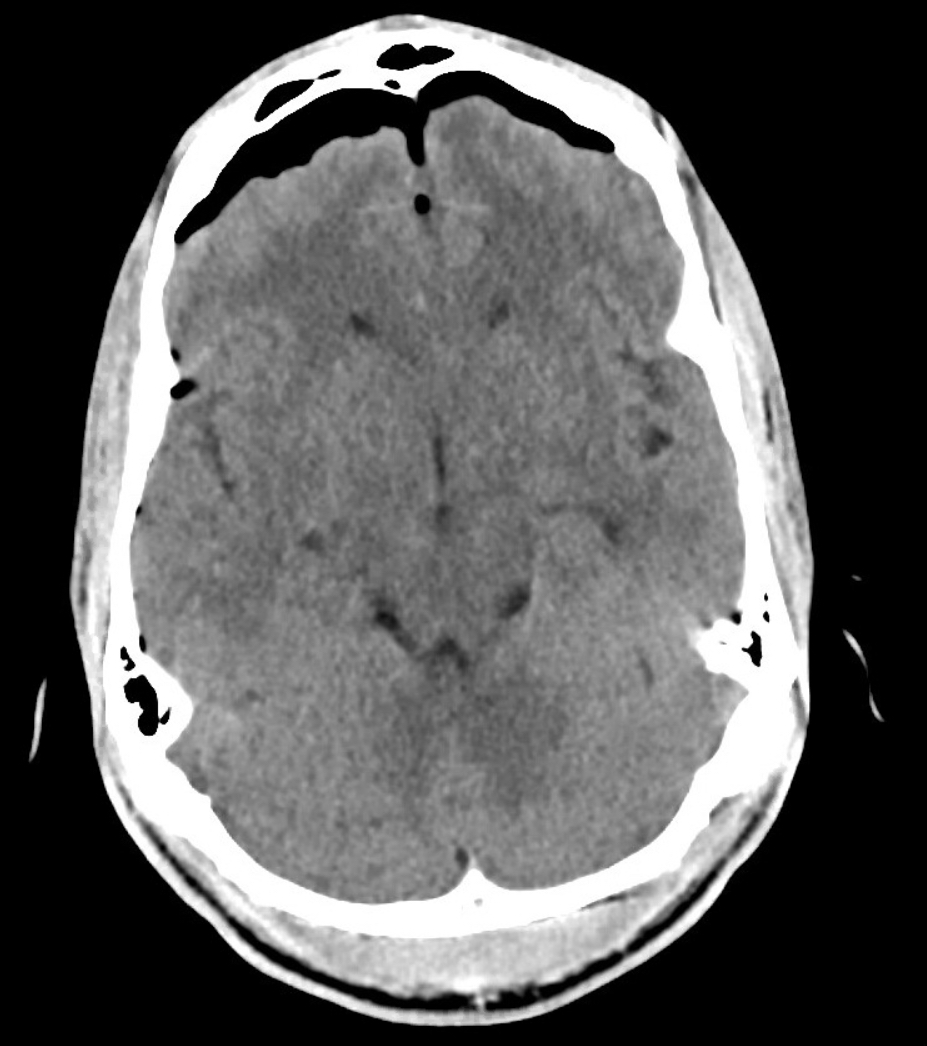

Supplement: Supplementary file 4 [file jetem-6-2-v13-supp4.jpg]
